# MicroRNAs of *Bombyx mori *identified by Solexa sequencing

**DOI:** 10.1186/1471-2164-11-148

**Published:** 2010-03-03

**Authors:** Shiping Liu, Dong Li, Qibin Li, Ping Zhao, Zhonghuai Xiang, Qingyou Xia

**Affiliations:** 1 The Key Sericultural Laboratory of Agricultural Ministry, College of Biotechnology, Southwest University, Tiansheng Road, Beibei, Chongqing 400715, PR China; 2 Beijing Genomics Institute, Beishan Road, Yantian District, Shenzhen 518083, PR China; 3 Institute of Agricultural and Life Sciences, Chongqing University, Shazhengjie, Shapingba, Chongqing 400030, PR China

## Abstract

**Background:**

MicroRNA (miRNA) and other small regulatory RNAs contribute to the modulation of a large number of cellular processes. We sequenced three small RNA libraries prepared from the whole body, and the anterior-middle and posterior silk glands of *Bombyx mori*, with a view to expanding the repertoire of silkworm miRNAs and exploring transcriptional differences in miRNAs between segments of the silk gland.

**Results:**

With the aid of large-scale Solexa sequencing technology, we validated 257 unique miRNA genes, including 202 novel and 55 previously reported genes, corresponding to 324 loci in the silkworm genome. Over 30 known silkworm miRNAs were further corrected in their sequence constitutes and length. A number of reads originated from the loop regions of the precursors of two previously reported miRNAs (bmo-miR-1920 and miR-1921). Interestingly, the majority of the newly identified miRNAs were silkworm-specific, 23 unique miRNAs were widely conserved from invertebrates to vertebrates, 13 unique miRNAs were limited to invertebrates, and 32 were confined to insects. We identified 24 closely positioned clusters and 45 paralogs of miRNAs in the silkworm genome. However, sequence tags showed that paralogs or clusters were not prerequisites for coordinated transcription and accumulation. The majority of silkworm-specific miRNAs were located in transposable elements, and displayed significant differences in abundance between the anterior-middle and posterior silk gland.

**Conclusions:**

Conservative analysis revealed that miRNAs can serve as phylogenetic markers and function in evolutionary signaling. The newly identified miRNAs greatly enrich the repertoire of insect miRNAs, and provide insights into miRNA evolution, biogenesis, and expression in insects. The differential expression of miRNAs in the anterior-middle and posterior silk glands supports their involvement as new levels in the regulation of the silkworm silk gland.

## Background

Following their initial discovery in worms, an increasing number of 18-30 nt-sized small RNAs have been identified as crucial regulatory molecules in multicellular organisms, animal viruses, and unicellular organisms [1-7]. Identification of abundant miRNAs and other small regulatory RNAs in different organisms is critical in improving our understanding of genome organization, genome biology, and evolution [8]. The silkworm, *Bombx mori *(*B. mori*), an important model organism used to investigate several fundamental biological phenomena (including development, gene regulation, and morphological innovation [9]), has been employed for silk production for about 5,000 years. The recently sequenced *B. mori *is the first lepidopteran insect genome that provides a resource for comparative genomics studies, facilitating our understanding of insect evolution [10]. The latest miRNA database release (miRBase 14.0) presents 91 silkworm miRNAs and two so-called miRNA* sequences originating from the RNA hairpin arm opposite the annotated mature miRNA-containing arm [2,11]. However, some of these miRNAs have been identified solely on the basis of sequence similarity to known orthologs, and have never been confirmed experimentally. Furthermore, the total number of silkworm miRNA genes is significantly lower than that in fruit fly (152) and human (701), and it is likely that further miRNAs remain to be discovered in the silkworm.

To extend the known repertoire of small regulatory RNAs expressed in the silkworm, we constructed and sequenced three small RNA libraries prepared from the whole body (WB) as well as the anterior-middle and posterior silk glands (AMSG and PSG) of day-3 fifth instar larvae. The silk gland of *B. mori *is differentiated into anterior, middle, and posterior sections [12,13]. Expression of all sericin genes is limited to the anterior and middle parts of the middle silk gland [14,15], whereas the fibroin genes are expressed exclusively in the posterior silk gland [16,17]. Both sericin and fibroin genes are topologically and temporally regulated at the transcriptional level in a concerted manner during larval development [18,19]. The spatial distribution of miRNAs may contribute to the mechanistic understanding of concerted silk protein synthesis. Each library was individually sequenced, and generated more than 5 million short reads, resulting in a total of 36 million reads, of which 1,819,103 were miRNA reads. The newly identified miRNAs significantly enhance our knowledge of insect miRNA species and provide insights into miRNA evolution, biogenesis, and expression in insects.

## Results

### Overall complexity of small RNA pools between the libraries

**Figure 1:**
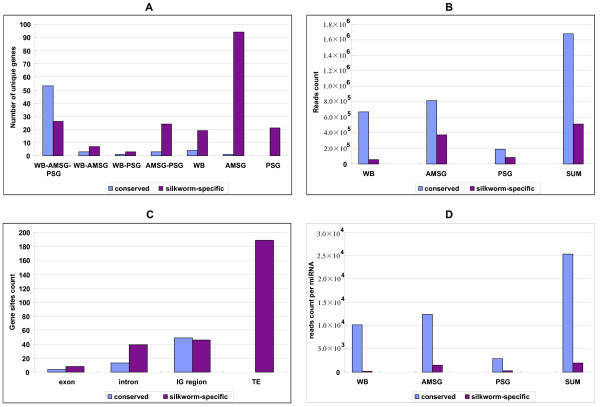
**Reads counts and genomic distribution of the conserved and silkworm-specific miRNAs**. (A) Detection of conserved and silkworm-specific miRNAs in the three libraries. (B) Genomic distribution of the conserved and silkworm-specific miRNAs. (C) Comparison of total reads count of the conserved and silkworm-specific miRNAs. (D) Averaged reads count per miRNAs. WB, whole silkworm body; AMSG, anterior-middle silk gland; PSG, posterior silk gland; WB-AMSG-PSG, across the three libraries.

We obtained raw data by sequencing three small RNA pools of the whole silkworm body from 5th-instar day-3 larvae, and anterior-middle and posterior silkworm silk glands, using the latest sequencing Solexa technology [8,20], filtered the low quality reads according to base quality value, trimmed the adaptor sequence at the 3' primer terminus, cleaned up 5' adaptor contaminants formed by ligation, and finally collected the small RNAs and analyzed size distribution. The raw data and processed files of the three libraries have been deposited in NCBI's Gene Expression Omnibus (GEO) [21,22] under accession number GSE 17965. For analysis, all identical sequence reads in each small RNA library were grouped and converted into unique sequences with associated counts of the individual reads. The flow results of data filtration for the three libraries are presented in Additional file 1. The total number of raw sequence reads in the whole body small RNA library is 5,467,768, comprising 2,848,263 low-quality reads (52.09%) and 2,619,505 high-quality reads (47.91%). The majority of high-quality reads in this library (95.65%) were longer than 18 nt, and used to map the silkworm genome assembly (build 2) using the SOAP program [23], leading to 1,844,635 genome-matched reads (73.78%). All clean reads of at least 18 nt were divided into different categories of small RNAs. The length distribution of high-quality reads was different among the three RNA libraries (Additional file 2). For example, some 778,343 (30.06%) sequences in the whole body are canonical 22 nt miRNAs, while 1,677,919 and 1,142,967 reads meet this length in the two silk gland libraries, accounting for 12.35% and 8.27% of the respective high-quality reads. A significant fraction of the clean reads was derived from putative degradation products of rRNAs, tRNAs, small nuclear RNAs, and other non-coding RNAs (27.38%). Another two large fractions were derived from unannotated genome sites (20.13%) and highly repeated sequences in the genome (20.57%). Substantial portions (2.58% and 2.18%) matched the exons and intergenic regions of protein-coding genes, respectively. In all, 680,406 reads (27.16% high-quality clean reads) were finally screened as miRNA candidates in the whole body small RNA library, and submitted for subsequent analyses. Similarly, the total sequence reads of potential miRNAs in the two silk gland libraries were 920,194 and 315,535, accounting for 7.78% and 2.04% of high-quality clean reads, respectively. Investigation of unique reads in the whole body and anterior-middle and posterior silk gland libraries revealed that the largest fractions were attributable to unannotated small RNAs (49.89%, 52.70% and 56.69% of high-quality clean reads, respectively). Upon addition of the known and unannotated miRNA loci, the unique sequence reads of candidate miRNAs in the three libraries were estimated as '923', '2,355' and '1,586', accounting for only 2.43%, 1.81%, and 1.98% of the respective high-quality clean unique reads. After successive filtering of these data sets, we identified 257 unique miRNA genes comprising 55 known and 202 novel genes, which collectively correspond to 324 independent genomic loci (Additional file 3). Notably, however, the majority of total or unique reads in this category in the three libraries were derived from annotated miRNA hairpins. The sequence reads and hairpin structures of all sequenced miRNAs in the whole body, anterior-middle silk gland, and posterior silk gland are detailed in Additional files, 4, 5, and 6, respectively. Reads counts and genomic distribution categories of the conserved and silkworm-specific miRNAs were summarized in Figure 1.

### The majority of known silkworm miRNAs are conserved across species

To date, only 94 miRNAs of *Bombyx mori *have been reported (Additional file 7) [24-30], and are available from latest miRBase database (Release 14.0) [31]. Some of these miRNAs were identified based solely on sequence similarity to known orthologous miRNAs, and have never been confirmed experimentally (Additional file 7) [24,26,32]. Here, we present evidence to support the authenticity of 55 known miRNAs. The remaining 39 known miRNAs, including bmo-mir-124 and bmo-mir-1926, were not successfully sequenced, possibly because of low expression levels or stage-/tissue-specific transcription. In total, 10 conserved miRNAs were firstly identified in the silkworm, 54 conserved miRNAs were sequenced in all three RNA libraries, while 12 were detected in only one or two small RNA libraries (Figure 1A; Additional file 7). For example, bmo-miR-133, miR-137, and miR-932 were identified in the whole body, but not in the anterior-middle and posterior silk gland, opposite to the patterns shown by bmomiR-285, miR-929, and miR-9b. Sequence comparisons between silkworm miRNA candidates and other miRNAs present in miRBase (miRBase 14.0) revealed that 23 silkworm miRNAs (including the new homolog of dme-miR-33) are widely distributed in over 20 species from invertebrates to vertebrates, 13 (including the novel homolog of lgi-miR-1175) are evolutionarily conserved throughout invertebrates, 32 (including 7 new sequences) exist only in the Insecta, and 37 known miRNAs (bmo-mir-1920, mir-1921, mir-1923, mir-1926 and 34 latest members) are presently confined to *B. mori *(Additional file 7). All conserved silkworm miRNAs were classified into known families or currently undefined groups on the basis of sequence similarity (Additional file 8). Various families of these miRNAs may have evolved for purposes as diverse as the phyla in which they occur. The identification of conserved miRNAs as potential phylogenetic markers raises the possibility that miRNAs serve as rapid evolutionary signaling molecules.

**Figure 2:**
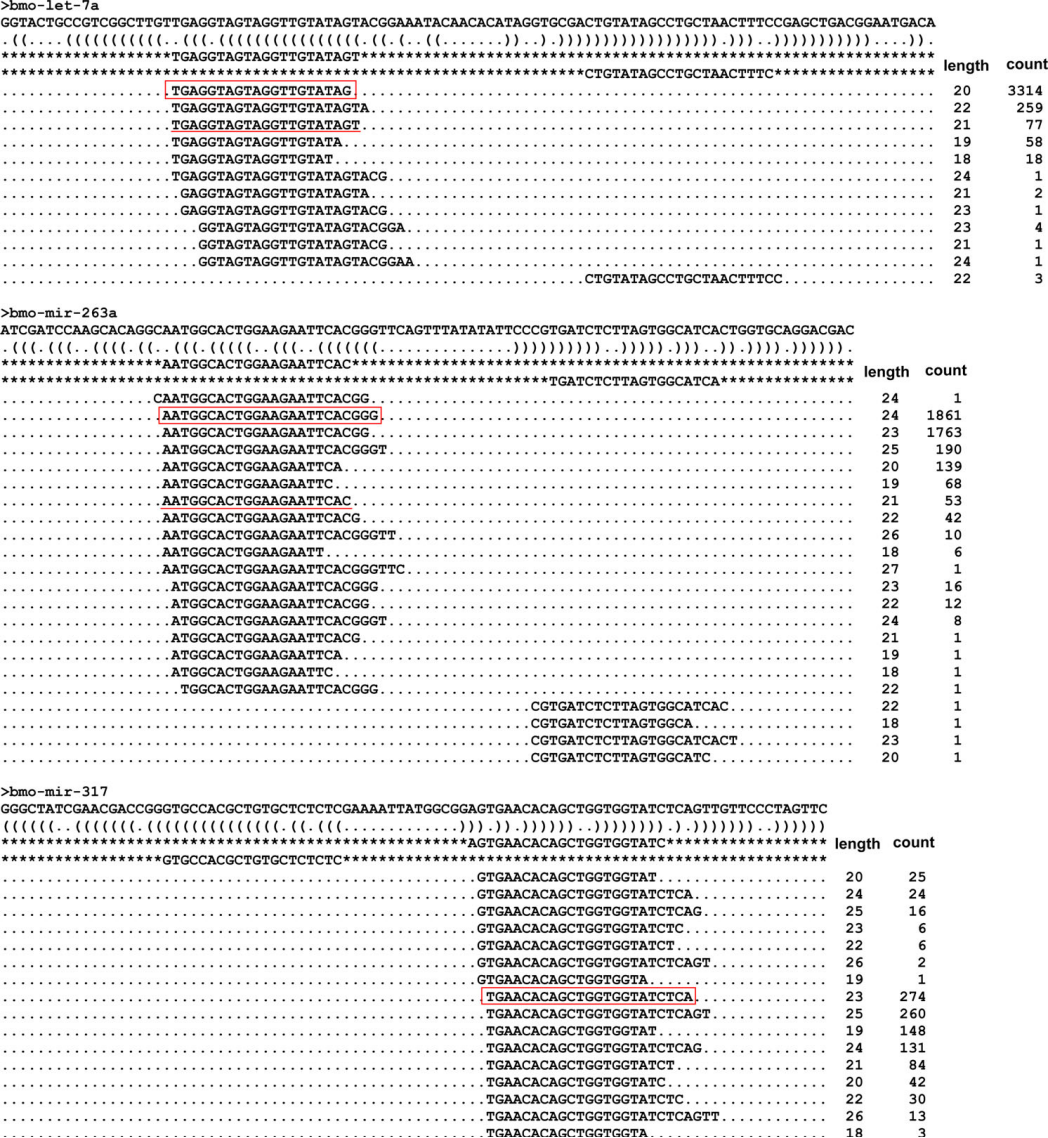
**Heterogeneity at the 5' and 3' ends of the sequenced tags**. Pre-miRNAs, structures, and multiple isoforms of expressed mature bmo-mir-263a, bmo-let-7a and bmo-mir-317 sequences and their read counts are shown. Underlined sequences are annotated in miRBase 14.0. The boxed sequences have the highest read counts. The annotated mature sequence of bmo-mir-317 was not identified, while the maximal sequence in the whole body was strongly accumulated in the two other libraries, and consequently regarded as the most active isoform.

**Figure 3:**
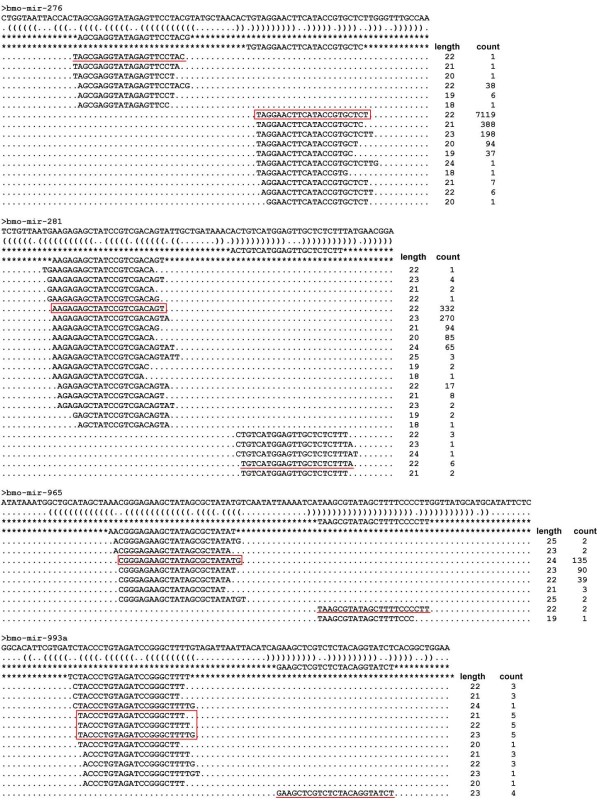
**Annotated miRNA and maximal sequences from different arms of the precursors**. The sequencing reads of four miRNAs in the whole body are depicted. Underlined sequences are the annotated mature sequences while the boxed sequences represent the maximum.

We observed heterogeneity at the 5' and 3' ends of sequenced tags derived from the same arms of the known miRNAs; strikingly, the 3' ends showed stronger heterogeneity than the 5' ends. As exemplified by bmo-miR-263a, let-7a, and miR-317 in Figure 2, the highlighted isoform of mature sequences is more highly accumulated in the three libraries than is the previously annotated sequence, and should be regarded as the final functional molecule. Similarly, over 30 known silkworm miRNAs were refined based on sequencing reads (Additional file 9). The miRBase annotations of silkworm miRNAs may thus be improved based on the most frequently sequenced miRNA isoforms. Nevertheless, some annotated miRNAs and highest reads were not derived from the same arms of the hairpin precursors, as illustrated by bmo-mir-10, bmo-mir-276, and bmo-mir-281 (Figure 3, Additional file 9). Accordingly, we postulate that these miRNA precursors produce functional molecules on both arms in one or more cell types in the silk gland or other tissues of the silkworm. Following identification by cloning [26], bmo-mir-1920 and mir-1921 exhibit sequential degradation from the 5' to 3' ends, and low levels of the annotated mature sequences accumulate in the three libraries (Additional file 10). Actually, a small number of reads originating from the loop regions exist in our sequencing data, but they should be by-products of processing. Well-established known miRNAs tend to show high numbers of reads for the miR and miR* sequences, and very small numbers or no reads elsewhere. Now, we find bmo-mir-1920 and mir-1921 suspicious with respect to being real miRNAs when we see significant numbers of overlapping reads tiling a whole region.

**Figure 4:**
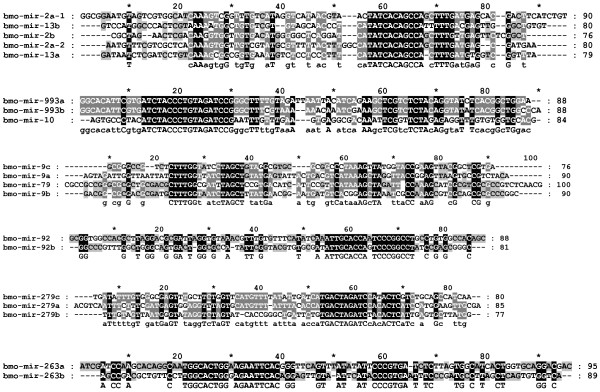
**Sequence alignment of precursors of conserved paralogous miRNAs**. The precursors were aligned using the CLUSTAL X program, and then highlighted with GeneDoc.

In all, 26 conserved miRNAs are closely clustered as polycistronic transcripts (<2.5 kb) (Additional file 11). Clusters 1-8 constitute conserved miRNAs, whereas clusters 9-12 comprise conserved and silkworm-specific miRNAs. All clustered miRNAs have different total read counts in any one of the three libraries. For example, the read counts of bmo-miR-275 are almost 10-fold greater than those of bmo-miR-305 in the anterior-middle and posterior silk gland, but only two-fold greater relative to the whole body. The majority of clustered miRNAs are derived from unique regions of the genome and, in general, are coordinately regulated. In cluster 6, bmo-miR-216 is intergenic, whereas other members of the cluster are intronic. The read count of bmo-mir-216 is far lower than those of intronic miRNAs in this cluster, and the miRNAs are thus unlikely to be coordinately regulated. Seven families of conserved miRNAs, including four clusters described above (2, 4, 7, and 8), have paralogs in the silkworm genome (mir-2, mir-993, mir-9, mir-92, mir-263, mir-279, and mir-989) (Figure 4). Significant differences were seen between the read counts of clustered paralog miRNAs across the three libraries (Additional file 12). In paralog 1, both arms of bmo-mir-10 produced over a 10-fold greater number of mature molecules than did bmo-mir-993a and mir-993b. Both mir-10 and mir-993b were confirmed in the three libraries, but mir-993a was absent in the posterior silk gland. The five members in paralog 2 or cluster 10 produced miRNAs from the 3' arms and star sequences from the 5' arms. However, neither miRNAs nor miRNAs* were equally accumulated in each library, and the star sequences of bmo-mir-2a-1 abnormally outnumbered miRNA sequences in the whole body. The read counts of bmo-miR-263a were at least 1000-fold higher than those of bmo-miR-263b in each library. The data collectively indicate that both paralogs and clusters are not prerequisites for the coordinate transcription and accumulation of miRNA genes.

### The miRNA depot of *Bombyx mori *is greatly enriched by newly identified miRNAs

In total, 209 unique miRNA and 87 star sequences were identified in this study (see Additional file 3). Sequence comparisons between the silkworm candidates and miRNAs of other organisms present in miRBase (miRBase 14.0) revealed that 11 novel silkworm miRNAs were orthologous to those identified in other species (Additional file 7) whereas most unique miRNAs displayed no evidence of evolutionary conservation, even within the Insecta, and thus appeared to be silkworm-specific (Additional file 3). The genome loci of all silkworm miRNA genes were characterized, as presented in Additional file 3 and Figure 1B. We identified 12 miRNA genes in the exons of protein-coding genes, including four conserved and eight silkworm-specific miRNAs. Four novel members, bmo-mir-2783, bmo-mir-2811, bmo-mir-2825, and bmo-mir-2829, were located within the introns and exons of single protein-coding genes. In total, 52 miRNAs, including 13 conserved and 39 silkworm-specific members were intronic, whereas 95 miRNA genes were widely distributed within intergenic regions (IG regions) of the silkworm genome. In contrast to conserved and known miRNAs, 190 silkworm-specific miRNA genes were located in predicted transposable elements, and were repeat-associated (Figure 1C, Additional file 3). Strikingly, these transposable elements are preferentially found outside protein-coding genes; only 30 of 181 unique transposable elements seem to be contained within predicted protein-coding genes. We identified 24 clusters consisting of 63 novel miRNA and 14 annotated genes. Clusters 3 and 4 were located in the second exon of the protein-coding genes, BGIBMGA010447 and BGIBMGA008683, respectively. Clusters 2, 7, 9, 16, 17, 18 were composed of intronic miRNA genes. Both clusters 6 and 12 consisted of intronic as well as intergenic miRNAs (Additional file 11), and are thus unlikely to be transcribed as polycistronic primary transcripts. Clusters 10, 11, 13-15 and 24 were located in intergenic regions, whereas five clusters (19 to 23) were derived from predicted transposable elements. We identified 45 groups of paralogs, 38 consisting of 110 silkworm-specific miRNA genes and 7 comprising 21 conserved miRNAs (Additional file 12). Paralogous miRNAs existed in 14 clusters, specifically, 2, 4, 7, 8, 13-15,, 18-24 (Additional file 11). These clusters possibly originated *via *a series of duplication and deletion events during silkworm evolution. The clustered paralogous miRNAs may have overlapping functions in regulating a similar set of genes, as reported for the three clusters, miR-106b~25, miR-106a~363, and miR-17~92 in mice [33].

A number of evolutionarily conserved miRNAs (let-7a, mir-1, and bantam) were among the most abundant miRNAs, as demonstrated previously [8,34-37], whereas the majority of novel miRNAs (particularly the silkworm-specific components) were among the least abundant (Additional file 13, Figures 1C,1D), consistent with the correlation between evolutionary conservation of miRNAs and their expression levels [35,38,39]. However, some conserved miRNAs displayed very low read counts; these included bmo-mir-282, bmo-mir-307, and bmo-mir-252. Some silkworm-specific miRNAs (bmo-mir-2755, bmo-mir-2756, bmo-mir-2758, and bmo-mir-2766) exhibited very high sequence reads, comparable to those of the abundant conserved miRNAs (Additional file 13). Nevertheless, momentary and local read counts cannot represent transcriptional levels during the entire life-cycle of the silkworm. For example, we could not identify bmo-mir-124 in the present study, but this miRNA was confirmed to be strongly expressed in the embryo and early larval stages of the silkworm [28]. Only 26 silkworm-specific miRNAs were common to the three libraries. In addition, 7 were shared by the whole body and the anterior-middle silk gland, 3 by the whole body and the posterior silk gland, 24 by the anterior-middle and posterior silk gland, 19 were in the whole body only, 94 were in the anterior-middle silk gland only, and 21 were identified only in the posterior silk gland (Additional file 13, Figure 1A). These data support the theory of several levels of complexity in miRNA processing and regulation in response to specific physiological circumstances.

## Discussion

The present results provide experimental evidence supporting the authenticity of 257 unique miRNA genes in the silkworm, including 202 novel and 55 known ones (Additional file 3). Analysis of the evolutionary conservation of all silkworm miRNAs revealed that only 23 were widely conserved from invertebrates to vertebrates, 13 were limited to invertebrates, and 32 had homologs in other insects (Additional file 7), whereas the majority of miRNAs were specific to the silkworm. Nearly 430 of the 447 newly identified chicken miRNAs were also likely to be exclusively expressed in chicken lineages [8]. These data indicate that most of the newly identified miRNAs are present in only a small group of organisms, and in some cases, in a single species [8,35-37]. Species-specific miRNAs may be large in number and evolutionarily dynamic as a result of gene duplication, sequence divergence and gene loss. A gray pawn hypothesis has been proposed for the species-specific chemoreceptor gene families in *Caenorhabditis *species [40]; this hypothesis suggests that individual genes are of little significance, but the aggregate activities of a large number of diverse genomic loci are required to establish a large phenotype space. The evolutionarily divergent miRNAs may also contribute to establishing and maintaining phenotypic diversity between different groups of organisms [41,42], and highly specific miRNAs have a specialized function in particular organisms, possibly involving regulation of lineage-specific pathways [8]. Furthermore, over 60% of the matched sequence tag fraction was attributed to unannotated small RNAs. The substantial proportion of unclassified small RNAs identified in this study may represent other classes of small regulatory RNAs in the silkworm that have not been covered in our analyses. Some of these may include rare miRNAs represented by very low sequence reads, thus not passing our filtering criteria. These candidates should be explored in future studies applying the deep sequencing approach to developmental stages, tissues, or cells.

Our sequence tag analysis led to the identification of miRNA and miRNA* sequences for 42 previously annotated miRNA genes and unilateral sequence tags for 11 known miRNA genes (Additional file 9). The total reads of 9 miRstar sequences (miR-10*, miR-276*, miR-281*, miR-282*, miR-2a-1*, miR-965*, miR-993a*, miR-993b*, and miR-9b*) were heavily skewed toward the RNA hairpin arm containing annotated miRNAs in at least one library (Additional file 9, names in green). Furthermore, we observed that bmo-miR-iab-4-5p levels exceeded those of bmo-miR-iab-4-3p in all three libraries (Additional file 9), thus revealing strand bias, even for the twin miRNAs. The reversal in the ratios of 5'- and 3'-derived sequence tags indicates preferential use of mature miRNAs originating from different arms of pre-miRNA precursors, and suggests additional levels of complexity in miRNA processing, which remain incompletely understood [8]. These findings are evidently inconsistent with the current knowledge of miRNA biogenesis and strand selection. The miRNA* strand is probably degraded rapidly on exclusion from the RNA-induced silencing complex (RISC), as the recovery rate of miRNAs* from endogenous tissues is 100-fold lower than that of miRNAs [43,44]. Consequently, in many cases, miRNA* cannot be detected using conventional methods, because of rapid turnover [8]. However, in the silkworm, a number of miRNA genes (mir-2a-1, mir-2b, mir-10, and mir-282) exhibited a similar number of sequence reads originating from the 5' and 3' arms of the miRNA hairpin precursors (5p and 3p, respectively) (Additional file 9*). The equivalent expression rates of miRNA and miRNA* largely result from similar 5' end stability that leads to equal incorporation of either strand into the RISC and protection from degradation [45]. In some cases, sequence tags originate from the terminal loop region of the pre-miRNA precursor (Additional file 10). Such examples have also been reported in other studies [46], and are explained as genuine products of pre-miRNA processing or random degradation products of unprocessed pre-miRNA [8].

**Figure 5:**
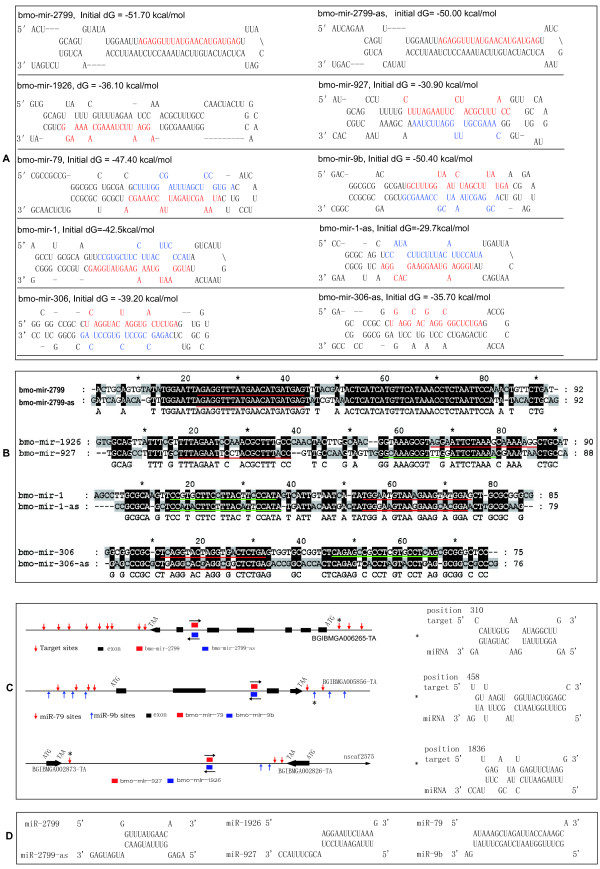
**Sense and antisense transcripts of silkworm miRNAs**. (A) Hairpin structures of the sense and antisense sequences of miRNA precursors. Mature miRNAs and miRNA stars are highlighted in red and blue, respectively. (B) Alignment of precursors. Mature miRNAand star sequences are underlined in red. (C) Schematic diagram of sense and antisense miRNAs and their potential target sites. Only one miRNA/target duplex proximal to CDS is presented (labeled with an asterisk). (D) Sense and antisense miRNAs target each other. All target sites were predicted using RNAhybrid2.2 and filtered with MirTif. The SVM score is higher than 1.0.

We identified 5 pairs of sense/antisense miRNAs in the silkworm (Additional file 3). For example, bmo-mir-927, -mir-79, and bmo-mir-2799-as are the reverse complements of the bmo-mir-1926, -mir-9b, and bmo-mir-2799 hairpins, respectively, and form hairpins reminiscent of miRNA precursors (Figure 5A). Sequencing tags from small RNA libraries of the anterior-middle and posterior silk glands mapped uniquely to the 5' arms of the bmo-mir-2799 and bmo-mir-2799-as hairpins. Interestingly, the sense and antisense transcripts display highly similar sequences, and are paralogous miRNAs (Figure 5B, Additional file 12). Their mature sequences are derived from complementary palindromes of the precursors. Both miRNA and miRNA* sequences were identified for bmo-mir-927 in the three libraries, but no sequencing reads mapped to the bmo-mir-1926 hairpin, although this has previously been identified in the silkworm moth [26], possibly indicating stage specificity. Sense and antisense miRNAs may coordinately control genes at their respective locations, as they both have target sites in the 3' untranslated regions (UTRs) and 5' UTRs of relevant genes (Figure 5C). The host gene BGIBMGA006265-TA is significantly enriched in all examined tissues of fifth-instar day-3 larval silkworm [47], and is optimally aligned to the thioredoxin domain of annotated *Homo sapiens *disulfide isomerase [48] that functions in protecting hypoxic cells from apoptosis [49]. Another sense/antisense miRNA pair, miR-79/miR-9b, also contains potential targeting sites for the untranslated regions of their home gene, BGIBMGA005856-TA, similar to the checkpoint kinase of *Mus musculus*. However, previous studies suggest that miRNAs do not target highly coexpressed genes [50,51], and thus it needs to be urgently determined whether the potential sites are indeed authentic and how expression of the sense/antisense pairs and their host genes are coordinated in biogenesis. The bmo-miR-927/miR-1926 pair is located in one intergenic region of the silkworm genome, but has target sites in the 3' UTRs of unannotated genes proximal to their loci. Significantly, the sense and antisense miRNAs target each other (Figure 5D). These miRNAs may potently downregulate each other via transcriptional interference, or may interact post-transcriptionally, or function *in vivo *via a feedback pathway. However, the issue of whether the sense and antisense transcripts are processed in the same cell type remains to be established, since sense and antisense miRNAs within an individual cell should interact. Genomic arrangement of two miRNAs arising from different strands of the same locus provides a simple and efficient means to create nonoverlapping miRNA expression domains [52-54]. The whole body and the anterior-middle and posterior silk glands comprise more than two cell types; a particular pre-miRNA may be processed into normal mature miRNA in one type of cell and miRNA* in another. While miRNA and miRNA* are equally processed, their functions may be distinct in the different cell types constituting this tissue. Therefore, the sequencing reads in unique cell lines of the silkworm should aid in determining the significance of miRNA, miRNA,* and sense/antisense transcripts.

## Conclusions

We have sequenced miRNAs in the whole body and silk gland of the silkworm. Our data confirm the authenticity of 257 miRNA genes in the silkworm, including 202 novel and 55 known miRNAs. Conservative analyses imply that miRNAs act as phyla markers in evolutionary signaling. The silkworm-specific miRNAs were significantly different between the anterior-middle and posterior silk glands. Target predictions revealed that the sense/antisense miRNAs target the 3' and 5' UTRs of their host genes. Identification of novel miRNAs resulted in significant enrichment of the repertoire of insect miRNAs and provided insights into miRNA evolution, biogenesis, and expression in insects.

## Methods

### Animal breeding and sample preparation

Domesticated silkworm (*Bombyx mori*) strain DaZao, was reared at 25°C on a natural diet of clean mulberry leaves. Silk glands were manually dissected from day-3 fifth larvae in 0.9% NaCl, rinsed with DEPC-treated water, and promptly immersed in liquid nitrogen.

### Small RNA library preparation and sequencing

Silk glands and whole bodies of day-3 fifth instar larvae were collected for RNA isolation. Following purification, total RNA samples were immediately preserved in ethanol and stored at -80°C until further use. For deep sequencing, the small RNA samples were prepared as follows: total RNA of each sample was size-fractionated on a 15% PAGE gel, and a 16-30 nt fraction was collected. The 5' RNA adapter (5'-GUUCAGAGUUCUACAGUCCGACGAUC-3') was ligated to the RNA pool with T4 RNA ligase. Ligated RNA was size-fractionated on a 15% agarose gel, and a 40-60 nt fraction excised. The 3'RNA adapter (5'-pUCGUAUGCCGUCUUCUGCUUGidT-3'; p, phosphate; idT, inverted deoxythymidine) was subsequently ligated to precipitated RNA using T4 RNA ligase. Ligated RNA was size-fractionated on a 10% agarose gel, and the 70-90 nt fraction (small RNA + adaptors) excised. Small RNAs ligated with adaptors were subjected to RT-PCR (Superscript II reverse transcriptase, 15 cycles of amplification) to produce sequencing libraries. PCR products were purified and small RNA libraries were sequenced using Solexa, a massively parallel sequencing technology.

### Computational analysis of sequencing data

Small 35 nt RNA reads were produced using an Illumina 1G Genome Analyzer at BGI-Shenzhen. Low quality reads were trimmed with our own perl script. Adaptor sequences were accurately clipped with the aid of a dynamic programming algorithm. After elimination of redundancy, sequences ≥ 18 nt were mapped to the silkworm Build2 genome using SOAP v1.11 [23]. Sequences that perfectly matched the genome along their entire length were considered for subsequent analyses. Genome sequences and annotations of the silkworm Build2 genome were downloaded from SilkDB [55]. Sequences matching silkworm rRNA, tRNA, snRNA and snoRNA deposited at the NCBI GenBank database or overlapping with rRNA and tRNA annotations of the Build2 genome were discarded. Repeat overlapping sequences were annotated as repeat-associated small RNAs. The majority of sequences overlapping with predicted exons were excluded from further analysis in view of the possibility that they were derived from messenger RNAs, and only those with precursors presenting p-values below 0.01 were collected.

After loading small RNAs and mapping information, small RNAs were sorted according to their position on the reference genome. Each read start position was examined to establish its similarity to a Drosha/Dicer processing site. For each candidate site, the mature sequence was extended to obtain two possible pre-miRNAs. One sequence encompassed 10 nt upstream and 70 nt downstream, and the other included 70 upstream and 10 downstream of the respective miRNAs. The two possible pre-miRNAs were evaluated using Mfold to determine ability to form characteristic hairpin structures [56]. A well-designed computational filter was employed to identify miRNA-like hairpins [57]. RNAs displaying folding energy values ≤ -25 kcal/mol were subjected to further analysis. Both miRNA and miRNA* had to reside in different arms of a hairpin structure, each with no more than 6 unpaired bases. We ensured that the maximum bulge over the miRNA/miRNA* duplex was not more than 4 bases, and asymmetry of the miRNA/miRNA* duplex equal to or less than 3. In addition to the above requirements, sequencing of both miRNA and miRNA* required that the miRNA/miRNA* duplex had 3' overhangs at both ends, a typical feature of Drosha and Dicer processing. Candidates corresponding to known miRNAs deposited at the miRBase 14.0 [11] and supported by two reads of mature sequences were considered real miRNA genes.

The criteria used to identify novel miRNAs from sequencing data of the three small RNA libraries were as follows: (1) genomic loci annotated as known silkworm miRNAs or other classes of noncoding RNA were excluded; (2) an individual locus had to be supported by a minimum of five independent sequence reads originating from at least one small RNA library to be considered for further analysis; (3) loci lacking hairpin-like RNA secondary structures, including positions of the small RNA tags, were eliminated. The resulting set of sequences and their respective RNA structures were further analyzed to distinguish genuine miRNA precursors from other RNAs containing similar RNA structures. All involved target sites were predicted using RNAhybrid2.2 [58] and filtered with MirTif [59]. The SVM score value was higher than 1.0.

## Abbreviations

BW: whole body; AMSG: anterior-middle silk gland; PSG: posterior silk gland; SilkDB: silkworm database; CDS: coding sequence; UTR: untranslated region; RISC: RNA-induced silencing complex; SVM: support vector machine.

## Authors' contributions

SPL conceived the study, reared and harvested all the samples, isolated small RNAs, analyzed the data, drafted and revised the manuscript. DL and QBL carried out the computational analysis. QYX and PZ participated in the design of the study and helped to draft the manuscript. ZHX coordinated the study. All authors read and approved the final manuscript.

## Supplementary Material

Additional file 1 **The flow results of data filtration and distribution of sequenced small RNAs across different categories**. After sequential filtration, the raw data in each library were separated into low and high quality reads. High quality reads with at least 18 nt were differentiated into categories of short sequencing reads, including rRNA, tRNA, snoRNA, fragments of sense and antisense exons and introns of coding genes, repeat sequences in transposable elements, unannotated short sequencing reads and some annotated miRNAs. Novel miRNAs were screened from unannotated sequencing reads and repeated sequences in transposable elements. Final miRNA data in each library are highlighted in blue. (A) Yield of whole body. (B) Yield of anterior-middle silk gland. (C) Yield of posterior silk gland. Click here for file

Additional file 2 **Distribution of clean tag reads from whole body and silk gland batches**. As shown in Additional file 1, the total high quality reads in each library (2,619,505 in the whole silkworm body) minus reads of adapters and inserts represent the clean tag reads in this Additional file. However, only sequences equal to and longer than 10 nt in each library were summed up for total reads in the respective tables (to the left of this file). Graphs on the right are derived from data from the table (left). Click here for file

Additional file 3 **Silkworm miRNAs identified by Solexa sequencing**. In all, 324 miRNA genes (loci) were identified in the silkworm genome, responsible for 209 unique miRNAs and 87 star sequences, which were screened from the raw data through flow data filtration. All known and novel miRNAs have been submitted to the international public DataBase, miRBase. The temporal names of novel miRNAs were replaced by the assigned names. The bmo-mir-276 well mapped to Build 1, but not to the Build2 genome sequence (blast 1e-5). Three new miRNAs, bmo-mir-100, bmo-mir-92b and bmo-mir-216, did not pass the filtering threshold due to their uncanonical hairpin structures, but were finally identified through homolog searches. Another two annotated miRNAs, bmo-mir-1920 and bmo-mir-1921, were not included in this file, since they showed sequential processing in generating mature sequences and no more accumulated reads were observed (Additional file 10). Several novel miRNA genes have duplicated loci within the silkworm genome. The U in the sequence was replaced with T, and sequences presented in the DNA form. Click here for file

Additional file 4 **Sequence reads and hairpin structures of all sequenced miRNAs in the whole body**. (A) Known silkworm miRNAs further identified using Solexa sequencing in the whole body. (B) Novel miRNAs of whole body predicted with mireap based on Solexa sequencing reads. (C) Repeat-associated novel miRNAs of whole body predicted as above. Click here for file

Additional file 5 **Sequence reads and hairpin structures of sequenced miRNAs in the anterior-middle silk gland**. (A) Known silkworm miRNAs further identified in the anterior-middle silk gland using Solexa sequencing. (B) Novel miRNAs of the anterior-middle silk gland predicted with mireap based on Solexa sequencing reads. (C) Repeat-associated novel miRNAs of the anterior-middle silk gland predicted using mireap based on Solexa sequencing reads. Click here for file

Additional file 6 **Sequence reads and hairpin structures of all sequenced miRNAs in the posterior silk gland**. (A) Known silkworm miRNAs in the posterior silk gland further identified using Solexa sequencing. (B) Novel miRNAs of the posterior silk gland predicted with mireap based on Solexa sequencing reads. (C) Repeat-associated novel miRNAs of the posterior silk gland predicted using mireap based on Solexa sequencing reads. Click here for file

Additional file 7 **Conservation analysis of silkworm miRNAs**. All known miRNAs and their orthologs were downloaded from miRBase 14.0. Abbreviations: inv-ver, widely identified in invertebrates and vertebrates; inv, identified in invertebrates; ins, identified in insects; sw, reported only in the silkworm. The colors in 'conservation' only highlight the differences in their conservation for easier viewing. Three miRNAs, bmo-mir-9b, bmo-mir-1924 and bmo-mir-1926, had no sequencing reads corresponding to previously reported miRNA sequences across the three libraries but the star sequence of bmo-mir-9b, miR-9b*, was identified in the anterior-middle and posterior silk glands (highlighted in blue). Nucleotides in red in some mature sequences were confirmed by Solexa sequencing. Click here for file

Additional file 8 **Insight into phylogenetic relationships among species from categorization of conserved silkworm miRNAs**. To know about phylogenetic relationships among diverse species on the basis of conservation of miRNAs, we used all known and novel silkworm miRNAs to BLASTN against the localized mature sequence data downloaded from the latest miRBase database. Only those miRNAs and miRNA*s with high sequence similarity to the mature sequences of silkworm are left in this file. The first miRNA in each group (highlighted in blue) is the silkworm miRNAs identified in this study or previously reported. Click here for file

Additional file 9 **Known silkworm miRNAs refined based on Solexa sequencing results**. Different nucleotides in sequences from miRBase 14.0 are highlighted in blue and different nucleotides in the Solexa sequencing reads highlighted in red. Annotated mature sequences of 9 miRNAs (green in the 'name' column) were significantly fewer than those from other arms of the hairpin precursors. The reported mature sequences of bmo-mir-1920 and bmo-mir-1921 (purple) should be refined on the basis of Solexa sequencing reads. Annotated sequences from miRBase 14.0 and sequences with the highest reads are presented in bold. Click here for file

Additional file 10 **Heterogeneity of mature sequences of bmo-mir-1920 and mir-1921**. (A) Heterogeneity in the whole body. (B) Heterogeneity in the anterior-middle silk gland. (C) Heterogeneity in the posterior silk gland. Click here for file

Additional file 11 **Closely clustered miRNAs of the silkworm**. For our analysis, groups of miRNA genes located within 2.5 kb of each other are defined as clusters. The close proximity of these miRNA genes in each cluster implies that they are likely to be transcribed as a single polycistronic transcript. Actually, more clusters can be identified when the space region is extended to a wider region than 2.5 kb. Click here for file

Additional file 12 **Paralogous miRNA genes of the silkworm**. Shown here are 45 groups of paralogous miRNA genes identified on the similarity of mature sequences, miRNAs or miRNA*s. Repeats of some miRNA genes in the genome are also considered paralogs since they are responsible for high similar (even identical) mature sequences. For the convenience of comparison and description, much information shown in Additional file 3 was also presented in this file. Click here for file

Additional file 13 **The reads count of conserved and silkworm-specific miRNAs**. The existence (+) or not (-) of miRNAs in the three libraries were clearly summarized in the samples' columns, WB, AMSG, PSG. Other information maintained only for the convenience of comparative analysis. Click here for file
